# Influence of diatomite and its base modifications on the self-adhesive properties of silicone pressure-sensitive adhesives

**DOI:** 10.1038/s41598-023-40958-0

**Published:** 2023-08-22

**Authors:** Adrian Krzysztof Antosik, Karolina Mozelewska, Marlena Musik, Piotr Miądlicki

**Affiliations:** 1 Faculty of Chemical Technology and Engineering, Department of Chemical Organic Technology and Polymeric Materials, West Pomeranian University of Technology, Szczecin, Pulaskiego 10, 70-322 Szczecin, Poland; 2 Faculty of Chemical Technology and Engineering, Engineering of Catalytic and Sorbent Materials Department, West Pomeranian University of Technology in Szczecin, Piastów Ave. 42, 71-065 Szczecin, Poland

**Keywords:** Engineering, Materials science

## Abstract

The study examined how diatomite and its modifications affected the self-adhesive ability of silicone pressure-sensitive adhesives. To create adhesive composition for testing, fillers were added to a commercial silicone resin, which were then used to create new modified pressure-sensitive tapes. The resulting tapes were tested to determine their adhesion, tack, cohesion at room and elevated temperature, SAFT test (Shear Adhesive Failure Temperature), pot-life (viscosity) and shrinkage. The results obtained were compared with those of the unmodified tapes. The tests resulted in higher thermal resistance (225 °C) and lower shrinkage (0.1%). As a result, we can conclude that materials with thermal resistance with a slight decrease in other parameters were obtained.

## Introduction

Pressure-sensitive adhesives (PSA) are relatively new materials in the rail industry. As "eternally alive" viscoelastic materials, they exhibit permanent tacky at room temperature in solvent-free state,—tack when lightly pressed and no liquid to solid transformation. PSAs come in many different forms—double-sided adhesive tapes of various thicknesses with backing or functional foam cores, self-adhesive transfer tapes, single-sided tapes, and printing foils. In addition to their various forms, pressure sensitive adhesives have different chemical formulations and are suitable for a wide range of industrial applications. Among all pressure-sensitive adhesives, there are three most popular chemical formulations of which they are obtainable—acrylates, silicones and rubber^1–4^.

Silicone pressure sensitive adhesives are referred to as specialty materials. Since their introduction in 1960, silicone pressure sensitive adhesives (Si-PSAs) have found many applications, including tapes for bonding low surface energy materials, as well as in the electrical and electronics, medical and healthcare, and automotive industries. High flexibility of Si–O–Si connections in self-adhesive silicone adhesives, low intermolecular interaction, low surface tension, excellent thermal stability and UV transparency, excellent electrical properties, chemical resistance, and resistance to weather conditions make these adhesives relatively better than other conventional carbon-based PSAs. Si-PSA with methyl groups and phenyl are cross-linked at a temperature between 120 and 150 °C with organic peroxides. The adhesives are inert and very hydrophobic while maintaining good water vapor permeability. Since 2000, there has been an increase in interest in new silicone pressure-sensitive adhesives; especially as medical and industrial tapes. More and more attempts are made to modify adhesives in order to increase the desired properties (e.g. thermal resistance). Due to the lack of functional groups in the polymer and our own research experience, the finished silicone resins are physically modified by adding a filler to increase the thermal resistance of the adhesive^5–8^.

Silicone tapes are best known for their ability to stick to low surface energy materials and resistance to a very wide temperature range—from − 40 °C to over 250 °C. The basic characteristics of this group of self-adhesive materials are the ability to form adhesive bonds with silicone materials, relative resistance to mold and fungus (because silicone is "inorganic", which means that the skeleton is non-carbon, it is hypoallergenic in nature). Disadvantages are often mentioned the relatively low bond strength with non-silicone materials^9–11^.

Researchers are working to significantly increase the potential of zeolite raw material, such as diatomite, through the use of various modification methods. Modification is of great interest mainly because of not only the significant occurrence and properties of such materials, but also the optimized yield and low cost. Modified zeolites represent versatile prospects for various applications. Modification of zeolites is most often used to increase their ion-exchange capacity, to increase their capacity and to obtain a more selective material^12^.

Diatomite is a material of sedimentary origin. The diatomite deposits consist mainly of an accumulation of skeletons formed by the formation of diatoms. The skeletons are amorphous hydrated or opalescent silica, but sometimes consist partly of alumina. Diatomite besides diatom shells usually contains other sediments such as clay and fine sand^13^.

Wang, Yang et al. modified the diatomite by easy alkaline treatment. The crude diatomite was dispersed in NaOH solution at 90 °C while stirring for 2 h. After separation, the modified diatomite was washed several times with water and anhydrous ethanol until a pH value close to neutral was obtained^14^.

Boriskov et al. modified the diatomite with 1N hydrochloric acid, and by using a 1N NaOH solution for 1 h was carried out. The samples were then washed repeatedly with distilled water, neutralized to pH 7. The next step was drying at room temperature to an air-dry state. Thermal activation was carried out by annealing the native, pre-washed and sieved diatomite in a muffle furnace for 3 h at 440–460°C^14^.

Xu et al. modified diatomite by KOH through mixed them in a round-bottom flask with a rotor at 60 °C for 24 h, with a KOH/diatomite mass ratio of 1:6 to 1:1. A magnetic heating stirrer was used to impregnate the dry mixture . The sample was then calcined in a muffle furnace at 300 °C to 700 °C for 4 h^15,16^.

Diatomite, sodium hydroxide and distilled water were mixed in a three-necked flask^17^. The flasks were sealed with stoppers to prevent evaporation of the solution. The flask was immersed in a water bath at a fixed temperature and the system was kept stirring for 3 h. The resulting mixture was filtered on filter paper and then washed with distilled water until no sodium ions could be detected in the solution.

The goal of work was modification of self-adhesive silicone resins with the fillers to obtained new silicone pressure-sensitive adhesives composition with increased thermal resistance. Fillers assist in the transfer of temperature through the material, thus increasing thermal resistance. Self-adhesive tapes were obtained from the prepared compositions. Diatomite was selected as a filler improving thermal resistance; the filler was also alkali-etched.

## Materials

The following materials were used to create this paper:

- Silicone adhesive from Dow Corning (USA)—resin DOWSIL™ 7358 (Q2-7358).
- Cross-linking compound was dichlorobenzoyl peroxide—DClBPO (Gelest – USA).
- Solvent was toluene from Carl Roth (Germany).
- Diatomite (diatomaceous earth) from Nanga (Poland) was used as a filler.

According to the manufacturer's assurances, the particle size of diatomaceous earth (diatomite) is to 10 µm. Based on the manufacturer's tests, the chemical composition of the filler was assumed as: SiO_2_ 93.0%; Al_2_O_3_ 2.0%; Fe_2_O_3_ 1.0%; CaO 0.3%; Na_2_O + K_2_O 2.5%; MgO 0.5%; TiO_2_ 0.2%; P_2_O_5_ 0.1%.

Dowsil 7258 is a solvent-base silicone pressure-sensitive adhesive dispersion of polydimethylsiloxane gum and resin (52–58%) in toluene/xylene.

### Filler modification

Diatomite samples were modified with NaOH and KOH solutions of different concentrations of 0.1 M, 0.5 M and 1 M for 6 h at 60 °C. For modification, 100 ml of the corresponding solution per 10 g of sample was used. The whole was placed in an ultrasonic bath. The resulting samples were centrifuged (7000 rpm), washed with deionized water to pH 7 of filtrate and dried at 85 °C for 24 h.

### Preparation of one-side self-adhesives tape

To prepare one-side self-adhesive tape the commercial resin was medicated by using 1.5 parts crosslinking agent wt. for 100 parts wt. resin. Then 0.1; 0.5, 1,0 or 3,0 wt% filler was added to a modified resin and mixed until a homogeneous composition was obtained. That obtained compositions were coated onto a 50 μm thick polyester film using a semi-automatic PSAT coater. The composition coated in this way was introduced into a Binder dryer (Binder GmbH, Germany) at a temperature of 110 °C for 10 min for solvent evaporation and thermal cross-linking. That obtained adhesives film with a weight of 45 g/m^2^ was secured with another layer of polyester film.

## Methods

### FT-IR

The FTIR spectra were made using a Nicolet 380 spectrometer from Thermo Electron Corporation (Waltham, MA, USA). The study used the ATR attachment with a diamond crystal. The measurement range was from 4000–400 cm^−1^ at a resolution of 4 cm^−1^.

### X-ray diffraction (XRD)

The XRD of the modified diatomite’s was obtained by an Empyrean PANalytical X-ray diffractometer (Malvern, UK) with a Cu lamp used as the radiation source in the 2θ 10–100° range with a step size of 0.026.

### Pot life

The pot life is defined as the period of time during which a given composition can be coated without problems. After a long storage time of the composition (mainly after the addition of the cross-linker), the viscosity of the composition increases until the so-called gel point^18^. The tests were performed with a Brookfield viscometer at room temperature at intervals, immediately after mixing and after 1, 2, 3, 5, 7 days, respectively. Pot-life was determined using a DV-II Pro Extra viscometer (Brookfield, New York, NY, USA).

Using a solvent analyzer (Radwag MAX 60/NP, Radom, Poland) was measured the solid’s content of the starting adhesive. The measurement was carried out in aluminum crucibles at 140 °C for 40 min. The basis weight of the adhesive film was measured using a round punch with area of 10 cm^2^ (Karl Schröder KG, Weinheim, Germany).

### Peel adhesion

Adhesion is a phenomenon of the interaction of particles, atoms and ions, adjacent surfaces. The measure of adhesion is the work per unit area that has to be done in order to release adhesively sticked materials^19^. The peel adhesion test was carried out in accordance with the AFERA 4001 test method on the Zwick-Z010 testing machine (Zwick/Roell, Germany). After removing the release liner, the foil was stuck to a steel plate using a 2 kg roller. The sample was left for 20 min. The peel adhesion was measured for each test piece at an angle of 180°. The result of the measurement is the average of the five measurements^20,21^.

### Cohesion

Cohesion is a type of intermolecular interaction due to which the molecules of a given substance are kept in close proximity. The measure of cohesion is the work required to separate a certain body into its parts divided by the area resulting from the separation. Cohesion depends, inter alia, on the state of aggregation, the microstructure of the material, and the size of interactions between molecules. Cohesion was measured according to the AFERA 4012 standard at 20 and 70 °C. According to this method, a part of the strip measuring 2.5 cm × 2.5 cm is stuck to the steel plate, and the other end of the strip is loaded with a weight of 1 kg. The test measures the time that elapsed until the adhesive film separates from the tile^22^.

### Tack

The ability to stick two surfaces quickly and reliably is a key performance requirement in many pressure sensitive adhesive products, especially adhesive tapes and labels. The key property for successful bonding is the tack or "stickiness" of the adhesive. The tack test is performed in accordance with the AFERA 4015 standard. The value of the tack is defined as the force required to separate, at a predetermined speed, the loop which touches an area with a predetermined surface by means of an adhesive^23,24^.

### SAFT test (shear adhesion failure temperature)

The samples were prepared according to the procedure described for the cohesion tests. A mass of 1 kg was suspended at each end of the sample and placed in the oven. The temperature was increased from 22 to 217 °C at a heating rate of 1 °C/min. The damage temperature was given along with the nature of the adhesive damage. Tests were carried out on 4 samples for each formulation to determine the mean temperature resistance, and the standard deviation was used as the error^23^.

### Shrinkage

Shrinkage was measured using the cross method, where the PVC film is coated with a thin layer of adhesive, cross-linked and then sticked to a metal plate. Two 90° incisions are made. Measurements are made by measuring the width of the cuts at various intervals keeping the test temperature at 70 °C. Shrinkage is a percentage of the ratios of the cut widths. Shrinkage greater than 0.5% is not allowed^20,25^.

## Results and discussion

An adhesive composition with good functional properties was selected for modification with fillers. The results for the pressure-sensitive adhesive without modification are presented in Table 1. However, self-adhesive tapes without modification show a low temperature value in the SAFT test, which limits the range of applications of such tapes. At the same time, they are characterized by good cohesion at room temperature and elevated temperature. Moreover, the adhesion and tack values are good. This leads to the conclusion that the planned physical modifications with diatomite based fillers can have a positive effect on the Si-PSA proportions. It was expected that this addition would improve the thermal properties of self-adhesive tapes while maintaining the performance properties, such as adhesion or cohesion, at the same level as unmodified tapes. The modified adhesive had a viscosity equal to 16.7 Pa s and contained 57.6% of solids (measured thermogravimetrically). Only the crosslinker (thermal)—1.5 pph of dibenzoyl peroxide was added to it and diluted with toluene (to 55% solids).

**Table 1:** Q2 7358 pressure-sensitive adhesive results without modification.

Peel adhesion [N/25 mm]	12.75
Cohesion in 20°C [h]	> 72
Cohesion in 70°C [h]	> 72
SAFT (°C)	192
Tack [N]	10.45

### FT-IR

**Figure 1:**
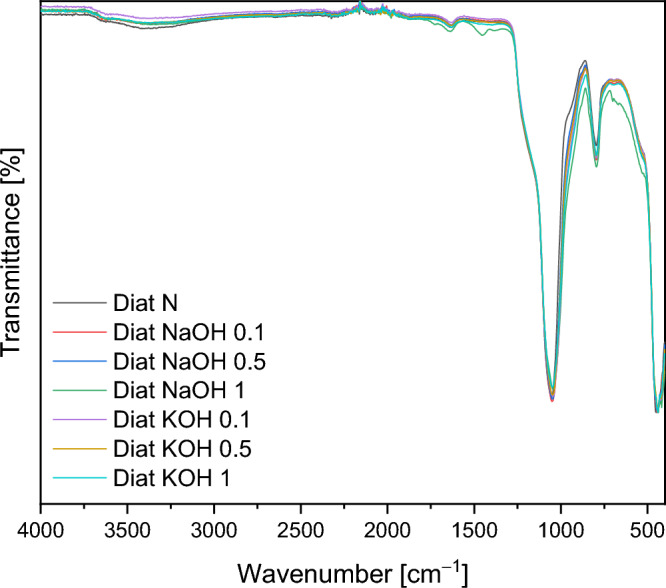
FTIR spectra of the raw diatomite and the modified diatomite’s NaOH and KOH solutions.

Figure 1 FTIR spectra of the raw diatomite and the modified diatomite’s are shown. The peaks at 3421, 1631 cm^−1^ and 960 cm^−1^, which are observed in all seven samples, are typically associated with stretching vibrations of the hydroxyl (–OH) groups on the surface of the samples. With increasing concentrations of NaOH and KOH the intensity of the peak at 960 cm^1^ increases, which is confirmed by the increase in the number of OH groups. The peaks at 1054, 796 and 451 cm^-1^ are attributed to asymmetric stretching, symmetric stretching and bending modes of the Si–O–Si bonds in the diatomite^26^.

### XRD

**Figure 2:**
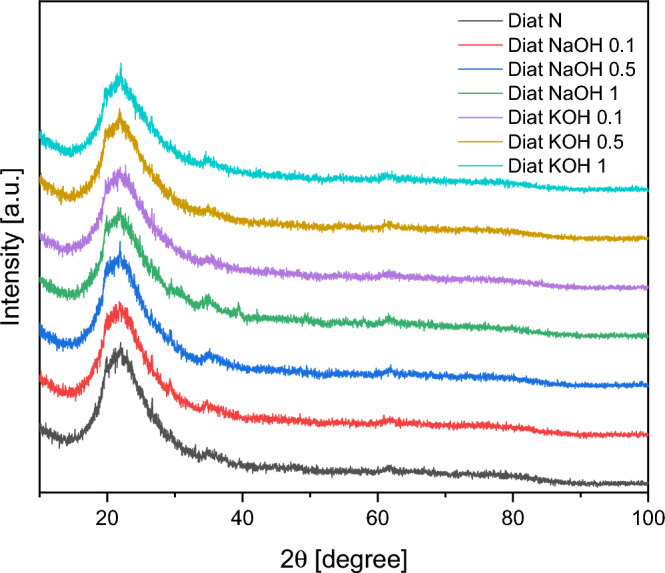
XRD of the raw diatomite and the diatomite’s modified with NaOH and KOH solutions.

Figure 2 shows the XRD patterns of the raw diatomite and the diatomite’s modified with NaOH and KOH solutions. A broad peak at about 22.5° associated with the amorphous structure of SiO_2_ is observed. These are typical non-crystalline diffraction peaks. The absence of other reflections indicates a very high purity of diatomite.

### Peel Adhesion

**Figure 3:**
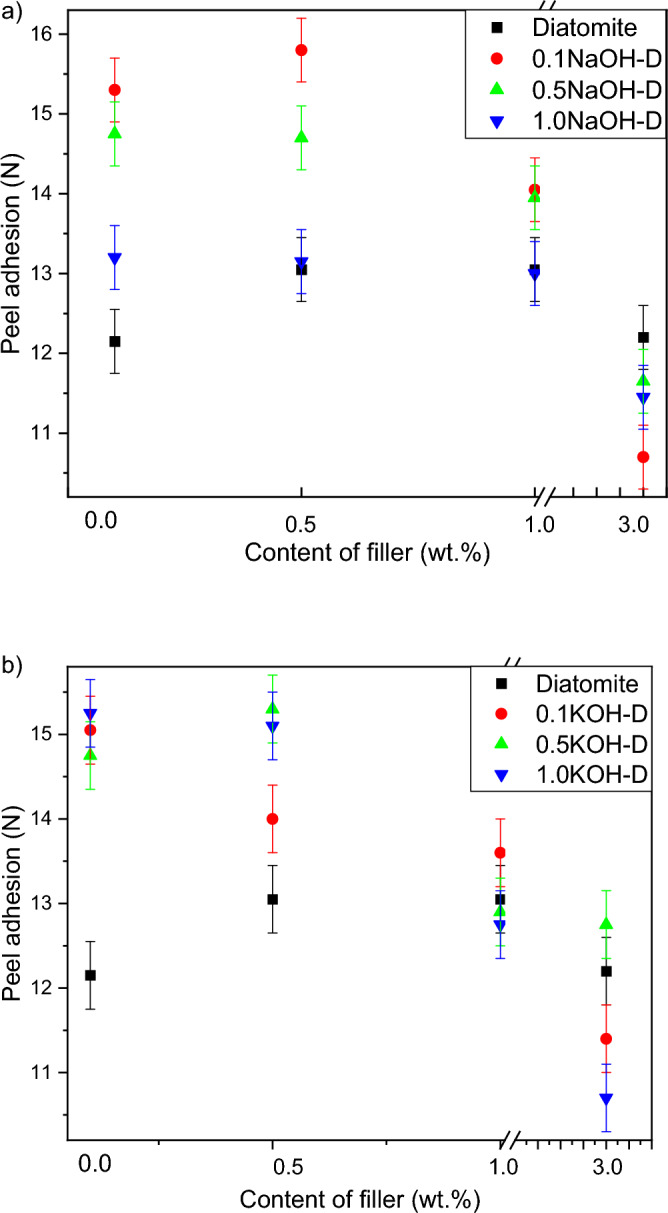
Effect of diatomite addition on the peel adhesion of silicone pressure-sensitive adhesives: (**a**) diatomite modified byNaOH; (**b**) diatomite modified byKOH.

Figures 3a and b show the effect of the addition of diatomite-based fillers on the adhesion of the adhesive film. The diagrams respectively show tapes modified with fillers etched with sodium and potassium base of different concentration in order to change the ratio of silicon to the rest of the filler. A small addition of the filler (0.1 or 0.5% by weight) increased the value of adhesion compared to the composition withoutthe filler, while with a large addition, its decrease was noted. It is caused most similarly by the effect of the desirable and more compact structure caused by the introduction of small amounts of filler, and when the amount is increased, it causes a shift of the cohesive-adhesive balance towards cohesion^27,28^. In each case of filler modification, an increase in adhesion value was observed compared to films modified with unmodified diatomaceous earth. The greatest increase was recorded for small fillings (0.1 or 0.5% by weight) with fillers modified with 0.1 and 0.5 molar solutions. Out of the samples modified with an additive 0.5 wt% filler the highest value of peel adhesivon obtained for diatomite modified 0.1 molar NaOH. − 15.8 N/25 mm. It was also noticed that even a small addition of a filler modified 1 molar base allows to achieve much higher adhesion than in the case of a use filler modified 0.1 molar base, where for the KOH-modified diatomite higher values were obtained by the system etched with a 0.5 molar base solution.

### Tack

**Figure 4:**
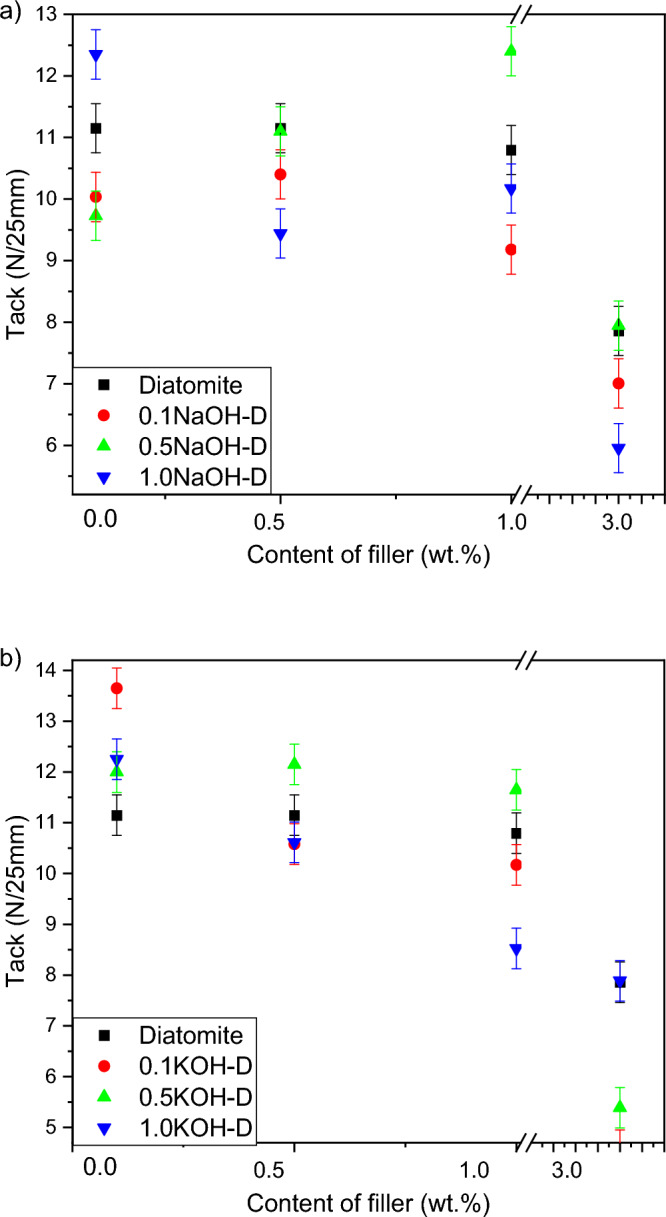
Efect of diatomite addition on the tack of silicone pressure-sensitive adhesives: (**a**) diatomite modified byNaOH; (**b**) diatomite modified byKOH.

The tack of the modified adhesive films maintained a similar tendency as in the case of its adhesion (Fig. 4). A small addition − 0.1% by weight of the filler increased the value of tack compared to the composition with the filler. Already for the addition of 1.0 wt. fillers, a reduction in tack value close to or lower than the value achieved by the unmodified adhesive film has been reported. This relationship is related in part to the shift of the cohesive-adhesive balance towards cohesion and the arrangement of the adhesive film. A systematic decrease in the value of stickiness after its increase may also be caused by the appearance of glue blockages (particles of the filler not covered with the adhesive layer) on the surface of the adhesive material, which will automatically lower the value of tack^27–29^.

### Cohesion

**Figure 5:**
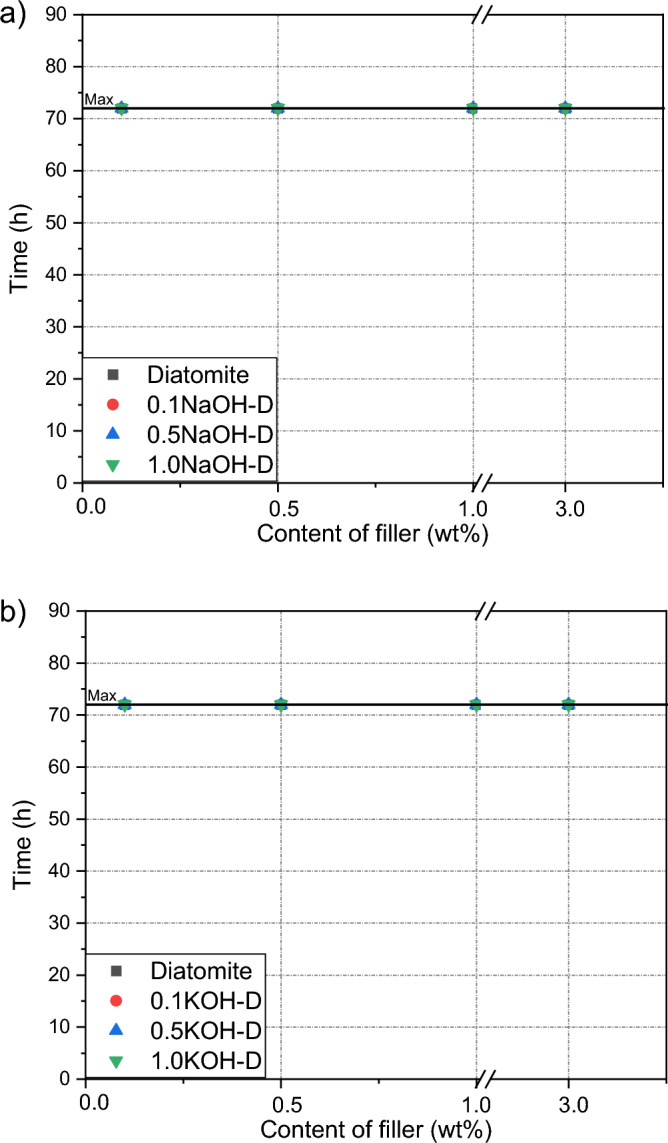
Efect of diatomite addition on the cohesion of silicone pressure-sensitive adhesives measurement at room temperature: (**a**) diatomite modified byNaOH; (**b**) diatomite modified byKOH.

**Figure 6:**
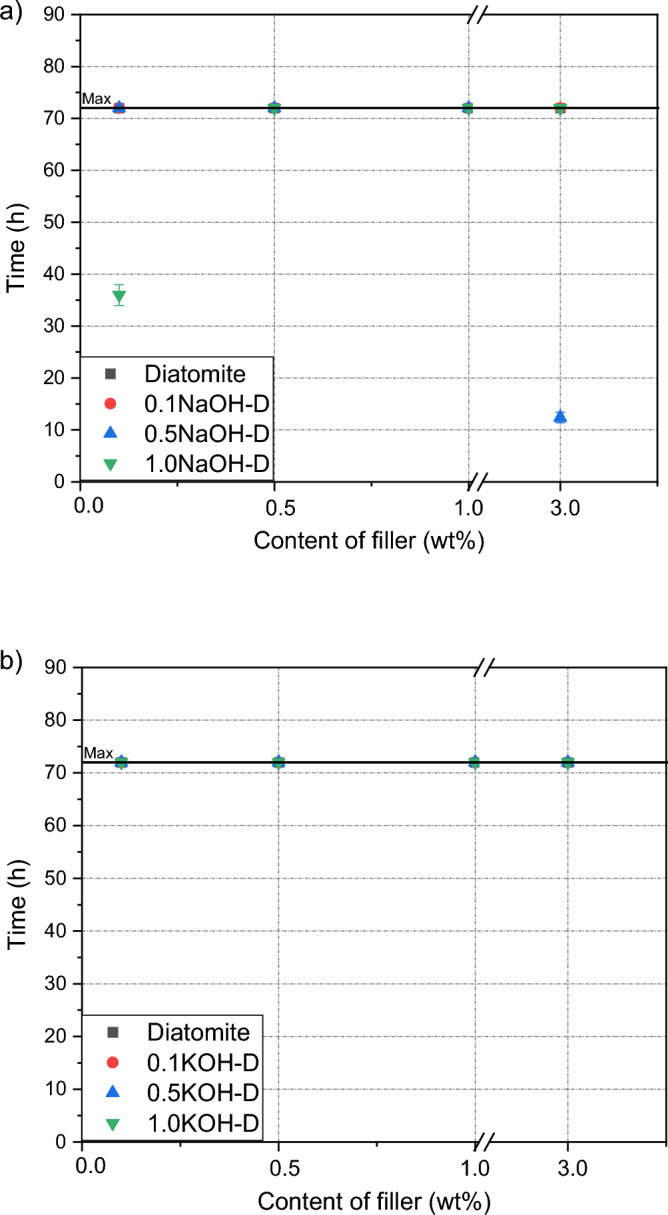
Efect of diatomite addition on the cohesion of silicone pressure-sensitive adhesives measurement at 70 °C: (**a**) diatomite modified byNaOH; (**b**) diatomite modified byKOH.

No effect of the filler addition or its basic modification on the cohesive properties of the adhesive composition was observed. In the case of both NaOH and KOH, all samples tested showed high cohesive resistance of over 72 h at room and elevated temperatures.. The test results are presented in Figs. 5 and 6. This effect confirms the good compatibility of the fillers with the silicone resin; and in combination with the results of adhesion and tackiness, a better arrangement of polymer chains and a more compact structure of the adhesive film caused by the addition of the filler are confirmed^27,28^.

### SAFT test

**Figure 7:**
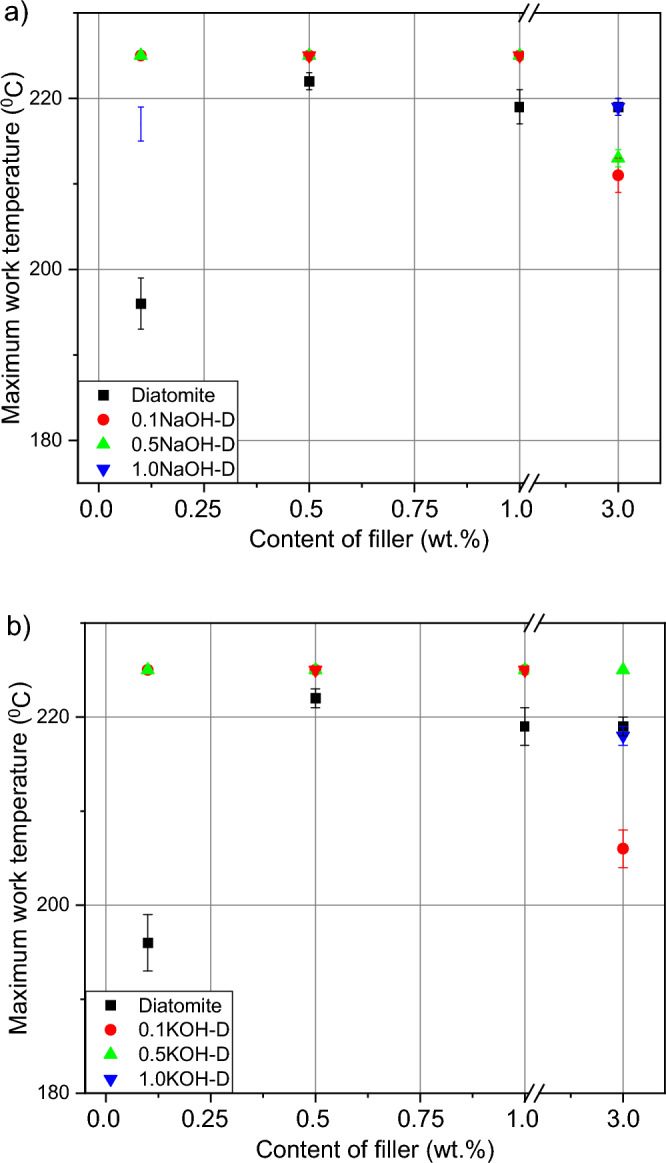
Efect of diatomite addition on the thermal resistance in SAFT test of silicone pressure-sensitive adhesives: (**a**) diatomite modified byNaOH; (**b**) diatomite modified byKOH.

The obtained new adhesive tapes were tested by means of the SAFT test in order to determine their thermal resistance and to compare them to unmodified tapes; the results are shown in Fig. 7. The addition of fillers increased the thermal resistance of the tested adhesive compositions, along with the increase of the additive, the temperature resistance increased each time, reaching the test limit of 225 °C in the region of 0.5 to 1.0% by weight of the additive. For samples etched with NaOH and KOH bases, this value was already achieved at 0.1 weight percent. At 3% by weight of the additive, a decrease in the maximum operating temperature was noted, but not below the value of the samples without fillers. Etching samples with bases improves their compatibility with silicone resin and increases its thermal resistance, it may be related to the increase in silicon mass in the pickled fillers compared to the rest of the filler^28–30^. Fillers assist in the transfer of temperature through the material, thus increasing thermal resistance^31^.

### Pot life

**Figure 8:**
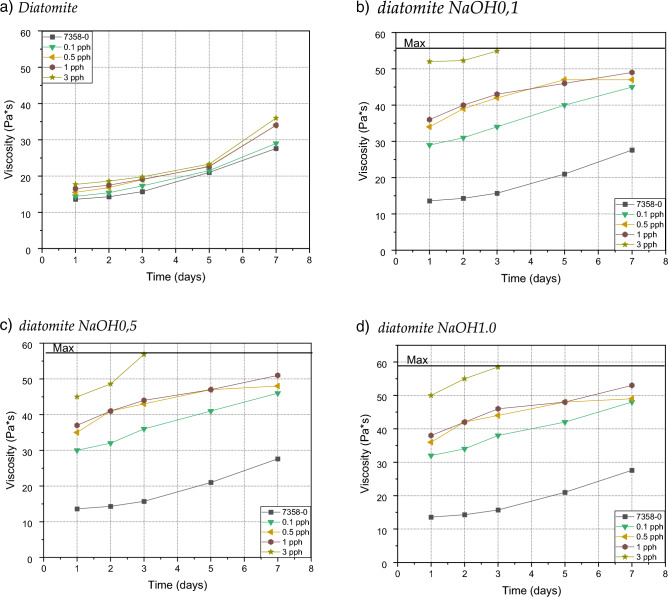

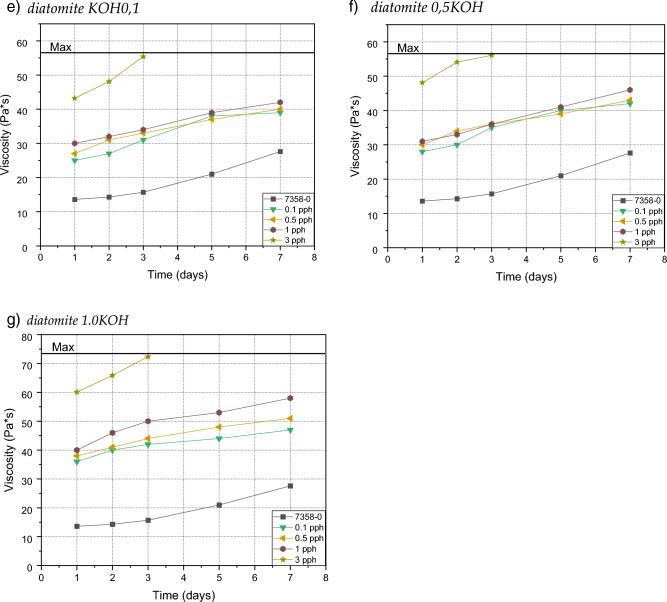
Efect of diatomite addition on the viscosity of silicone pressure-sensitive adhesives: (**a**) diatomite; (**b**) diatomite modified by 0.1 NaOH; (**c**) diatomite modified by 0.5 NaOH; (**d**) diatomite modified by 1.0 NaOH; (**e**) diatomite modified by0.1 KOH; (**f**) diatomite modified by 0.5 KOH; (**g**) diatomite modified by 1.0 KOH.

In each test case, the viscosity of the samples increased rapidly to form a gel—the samples doubled their viscosity or reached the maximum viscosity allowing the use of the adhesive composition for the 74 Pa s coating; in the first case, the samples had settled to the edge of the gel after about 7 days, while in the second case, the maximum was reached by samples with the addition of 3% by weight of alkali-modified filler, regardless of the molar concentration used for its modification. Compared to pure adhesive compositions, the addition of diatomaceous earth slightly affected viscosity, even at the highest additive level tested.. In addition, studies have shown that when modifying by using base, the distances between the individual curves in the graph expand with the amount of filler added. A slight addition of 0.1 weight percent of the modified filler increases the viscosity twice as compared to the viscosity of the composition with the addition of the unmodified filler. The difference between the addition of the modified filler 0.1, 0.5 and 1.0 is relatively close and oscillates around a twofold increase in viscosity, while with the addition of 3% by weight of the filler, about fourfold increase in viscosity was noted—Fig. 8. There were no particular differences after the modification with the base of NaOH and KOH. A key role is played by OH groups, which cause the carbon atoms of the benzene group to attract hydroxyl protons through OH-π interactions. Increased content of OH groups in the modified filler significantly increases viscosity^32^.

### Shrinkage

**Figure 9:**
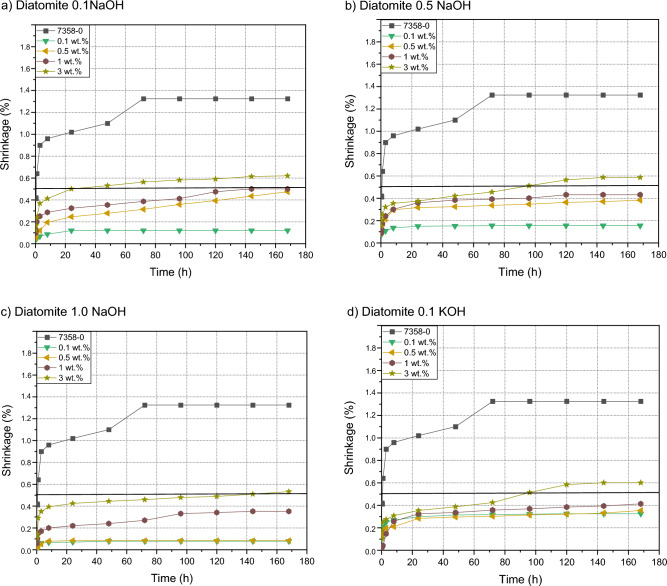

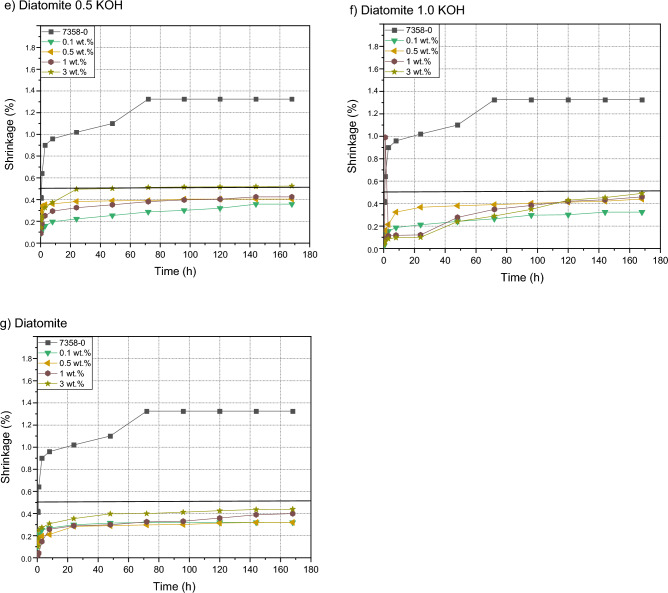
Efect of diatomite addition on the shrinkage of silicone pressure-sensitive adhesives: (**a**) diatomite modified by 0.1 NaOH; (**b**) diatomite modified by 0.5 NaOH; (**c**) diatomite modified by 1.0 NaOH; (**d**) diatomite modified by 0.1 KOH; (**e**) diatomite modyficated 0.5 KOH; (**f**) diatomite modified by 1.0 KOH, (**g**) diatomite.

Figure 9 shows the effect of diatomite and its alkaline modifications on the shrinkage value of pressure-sensitive silicone adhesives. The incorporation of a filler into the composition led to a dramatic reduction in the shrinkage value compared to an adhesive composition without fillers. The lower the addition of the filler, the smaller the skin of the tested samples was, the best results were obtained for fillings from 0.1 to 0.5% by weight. Most likely, this was due to a better alignment of polymer chains and a more compact structure, which improved the shrinkage of the adhesive film^28,33^. Modification of diatomite with bases allowed to obtain lower shrinkage values for each degree of filling of the adhesive compositions. The research showed that the materials treated with the highest molar concentrations gave the best results. The most probable reduction in shrinkage in this case was due to the increase in the proportion of silicon in the filler mass and the possible changes in its structure caused by the action of bases^29^.

## Conclusion

Research has resulted in self-adhesive tapes with improved properties compared to the starting adhesive. A very large increase was observed in the case of thermal resistance—most of the samples reached the maximum value: 225 °C. Shrinkage results were also exceptional, with virtually every filler addition resulting in a reduction in shrinkage. In each tested case, a rapid increase in the viscosity of the samples to the form of a gel was noted, so the obtained compositions are not suitable for storage and must be coated immediately after being tapped. In addition, the tapes retained good sub-aquatic properties such as adhesion, cohesion and tack, which makes them usable.

By introducing diatomaceous earth into the polymer matrix, new self-adhesive tapes with increased thermal resistance were obtained. Additional modification of diatomite with different bases and their introduction into Si-PSA resulted in further expansion of the possibilities of obtaining tapes with specific limiting parameters, thanks to which several self-adhesive products with specific thermal resistances can be prepared from the obtained results.

Wherever resistance to high temperatures and material expansion are required, the materials obtained can certainly be used. The main industry that could be the recipient of the products described in the paper is heating plants, where the tapes could be used to seal pipes.. In addition, these materials can be an interesting alternative to quick connectors used in the automotive industry, especially since they can be used in engines.

## Data Availability

The datasets used and/or analyzed during the current study available from the corresponding author on reasonable request.
